# Proposal for a Robot Rehabilitation Strategy Tailored to the Recovery Stage of Gait Impairment After Subacute Stroke: A Preliminary Case Report

**DOI:** 10.7759/cureus.117654

**Published:** 2026-10-08

**Authors:** Fumiaki Tamiya, Takaki Tateishi, Shouta Shimoda, Hiroshi Hosokawa, Ren Fujii

**Affiliations:** 1 Department of Rehabilitation, Musashigaoka Hospital, Kumamoto, JPN; 2 Musashigaoka Clinical Research Center, Musashigaoka Hospital, Kumamoto, JPN; 3 Department of Rehabilitation Medicine, Yamaga Hot Spring Rehabilitation Hospital, Kumamoto, JPN

**Keywords:** gait pattern optimization, hemiplegia, robot-assisted gait training, robot rehabilitation, stiff-knee gait, stroke rehabilitation, subacute stroke

## Abstract

Robot-assisted gait training (RAGT) has been increasingly applied as an intervention method for stroke hemiplegia patients' abnormal gait patterns. We describe an intervention strategy that attempts the precise adjustment of tasks' difficulty according to the severity of gait impairment by progressively adapting two types of robots in accordance with the subject's recovery process. This case report describes a 53-year-old man who presented with left hemiplegia due to a right lacunar infarction. He was admitted to our rehabilitation hospital 14 days after stroke onset and underwent a staged gait-training program from days 14 to 77 after stroke onset, including treadmill-driven RAGT from days 16 to 28 and orthosis-mounted RAGT from days 55 to 69. At his initial assessment, the patient required assistance with walking due to moderate motor paralysis. In the early stages of intervention, a treadmill-driven robot was introduced with the goal of improving the patient's walking independence. Subsequently, after supervised walking had been achieved and residual gait abnormalities persisted during conventional gait training, the patient transitioned to flat-ground walking practice using an orthosis-mounted robot with the goal of optimizing walking patterns. The sequential intervention was associated with improvement in walking independence, with the Functional Ambulation Category increasing from 1 to 5 by discharge, and improvements in stiff-knee gait and toe clearance were also observed. These findings suggest that stage-specific selection of robotic devices according to therapeutic goals may be a feasible approach for addressing both walking independence and residual gait abnormalities after stroke.

## Introduction

Approximately 80% of stroke patients with hemiplegia (hereinafter referred to as "hemiplegic patients") experience an impairment of their gait due to physical functional deficits such as unilateral motor paralysis, sensory deficits, and/or abnormal muscle tone [1]. Such gait impairments can be a major factor contributing to a significant decline in patients' activities of daily living (ADLs) and quality of life (QOL), making these impairments a primary disability that should be addressed through rehabilitation [2].

A crucial indicator for evaluating the potential gait impairments and walking ability of hemiplegic patients is the patients' walking speed, which strongly influences their walking independence and community mobility [2,3]. However, even after hemiplegic patients achieve independent walking within the normal speed range, they may show abnormal gait patterns involving asymmetry that often persist [4]. The abnormal gait patterns include knee hyperextension during the stance phase [5] and foot drop during the swing phase [6] and are associated with an increased risk of falls due to stumbling and the development of compensatory movements to counteract the abnormal patterns [7]. Another common abnormal pattern is stiff-knee gait, characterized by insufficient knee flexion during the swing phase, which can reduce toe clearance, defined as the vertical clearance of the foot above the walking surface during swing, and promote compensatory movements [7]. It is thus critically important to provide interventions that target abnormal gait patterns as part of the gait rehabilitation for individuals with hemiplegia, rather than focusing solely on their walking speed or independence level. Walking independence and gait-pattern normalization represent related but distinct rehabilitation outcomes. Improvement in functional ambulation does not necessarily indicate normalization of lower-limb kinematics; therefore, quantitative evaluation of residual gait abnormalities becomes particularly important once supervised or independent walking has been achieved. Rehabilitation robots (hereinafter referred to simply as "robots") have been introduced to address several rehabilitation targets, including gait dysfunction and abnormal gait patterns. For example, robot-assisted gait training (RAGT) has gained attention as an intervention for post-stroke gait dysfunction because it enables intensive, repetitive, and task-specific gait practice and has been investigated for improving walking ability, balance, and selected kinematic gait parameters [8].

The robots that are currently used for RAGT are classified into two types: treadmill-driven robots and orthosis-mounted robots. In the present case, the treadmill-driven system combined treadmill walking with a robotic long-leg orthosis that provided adjustable assistance to the paretic lower limb, whereas the orthosis-mounted system consisted of a robotic unit attached to a conventional long-leg orthosis and provided targeted assistance to knee motion during overground walking. The treadmill-driven robots have been reported to be useful for improving walking independence, and orthosis-mounted robots have been found to be effective for correcting abnormal gait patterns [9]. In the present strategy, the treadmill-driven robot was used primarily to support intensive gait practice aimed at improving walking independence during the early recovery phase, whereas the orthosis-mounted robot was used during overground walking to provide more focused assistance to knee motion after supervised walking had been achieved. After supervised walking was achieved, residual stiff-knee gait, characterized by insufficient paretic knee flexion during the swing phase, reduced toe clearance, and compensatory movements to secure foot clearance, was considered a barrier to further progression toward independent walking. The therapeutic focus therefore shifted from improving walking independence to modifying the residual gait pattern, and the orthosis-mounted robot was introduced to promote repetitive motor learning of a more normal-like swing-phase knee-flexion pattern during overground walking. Based on these differences in training context and therapeutic goals, we considered that combining different types of robots according to the patient's recovery stage could provide a clinically meaningful strategy.

This case report illustrates the progress of our approach, which involves the use of a treadmill-driven robot for early independent walking acquisition and an orthosis-mounted robot for learning flat-ground walking movements, in a phased manner. This approach allows the implementation of walking practice with difficulty levels that are adjusted for each specific objective, with the ultimate goal of achieving independent and natural flat-ground walking.

## Case presentation

The patient was a 53-year-old man who had experienced weakness on his left side. The following morning, he had worsening dysarthria and muscle weakness, prompting a visit to his primary care physician. A suspected cerebral infarction led to the patient's hospitalization. Magnetic resonance imaging revealed a right lacunar infarction involving the corona radiata and basal ganglia (Figure 1). The day of stroke onset was defined as day 0 throughout this report.

**Figure 1:**
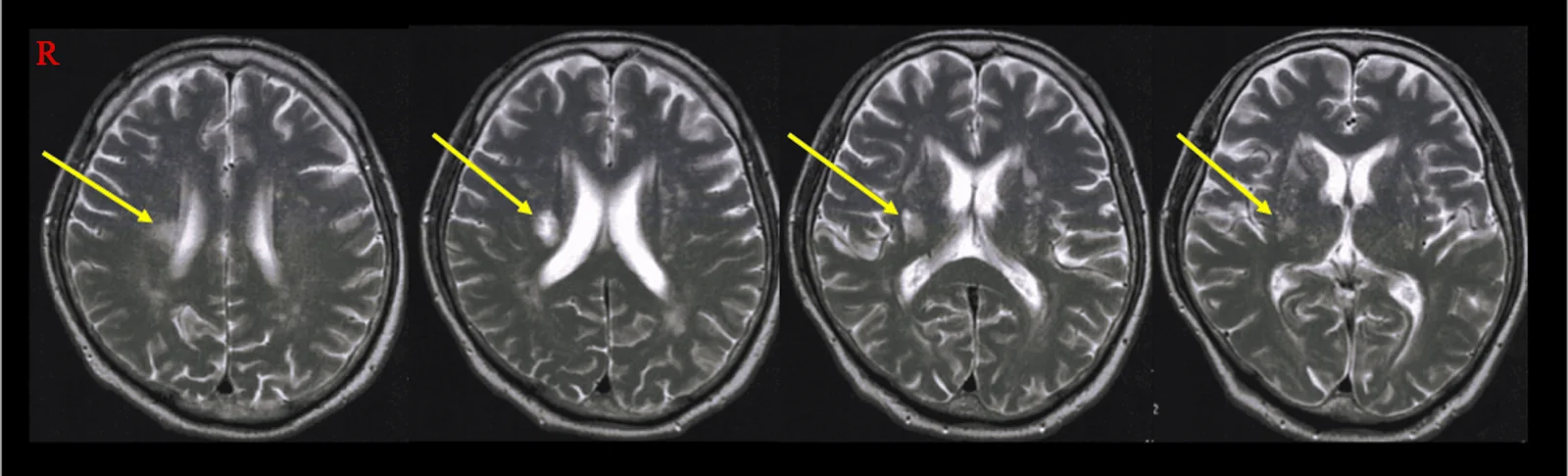
T2-weighted magnetic resonance images of the patient, a 53-year-old man The yellow arrows indicate the high-intensity lesion observed on T2-weighted magnetic resonance imaging. The lesion corresponds to a right lacunar infarction involving the corona radiata and basal ganglia.

Fourteen days after stroke onset, the patient was transferred to our hospital to continue rehabilitation. An intensive rehabilitation program was initiated, including physical therapy, occupational therapy, and speech therapy. At admission, the patient presented with moderate motor impairment of the left lower limb, with Stroke Impairment Assessment Set (SIAS) lower-limb motor scores of 3, 2, and 1 for hip flexion, knee extension, and foot-pat tests, respectively. Superficial and deep sensory scores of the affected lower limb were 3 and 2, respectively. Relevant vascular risk factors included hypertension and a history of smoking.

His walking ability was limited to assisted ambulation (Functional Ambulation Category (FAC): 1), and the Functional Independence Measure (FIM) motor score was 33. At admission, the patient required physical assistance for walking and demonstrated a markedly impaired gait pattern; however, detailed quantitative characterization of the abnormal gait pattern was performed later, after supervised walking had been achieved. The patient's FIM cognitive score at admission was 23 points, indicating impaired cognitive function; however, no higher-level cognitive impairments (e.g., unilateral spatial neglect, apraxia, or attention deficits) were observed. This cognitive impairment did not affect the patient's ability to understand instructions or participate in gait training. His cognitive function improved during hospitalization, and the FIM cognitive score increased from 23 at admission to 35 at discharge. Because no intermediate FIM cognitive assessments were available, the temporal relationship between cognitive improvement and the robotic interventions could not be determined.

Physical therapy intervention

The physical therapy intervention provided as part of the patient's intensive rehabilitation program consisted of active neuromuscular facilitation and lower limb/trunk muscle strengthening in the early stage for the paralyzed side, along with ADL practice and gait training. His gait training aimed for independent walking indoors and outdoors, combining general gait training with RAGT (Figure 2A). Conventional rehabilitation was continued throughout the hospitalization, while RAGT was incorporated at specific stages of recovery according to the therapeutic goals, in combination with conventional gait and orthotic training.

**Figure 2:**
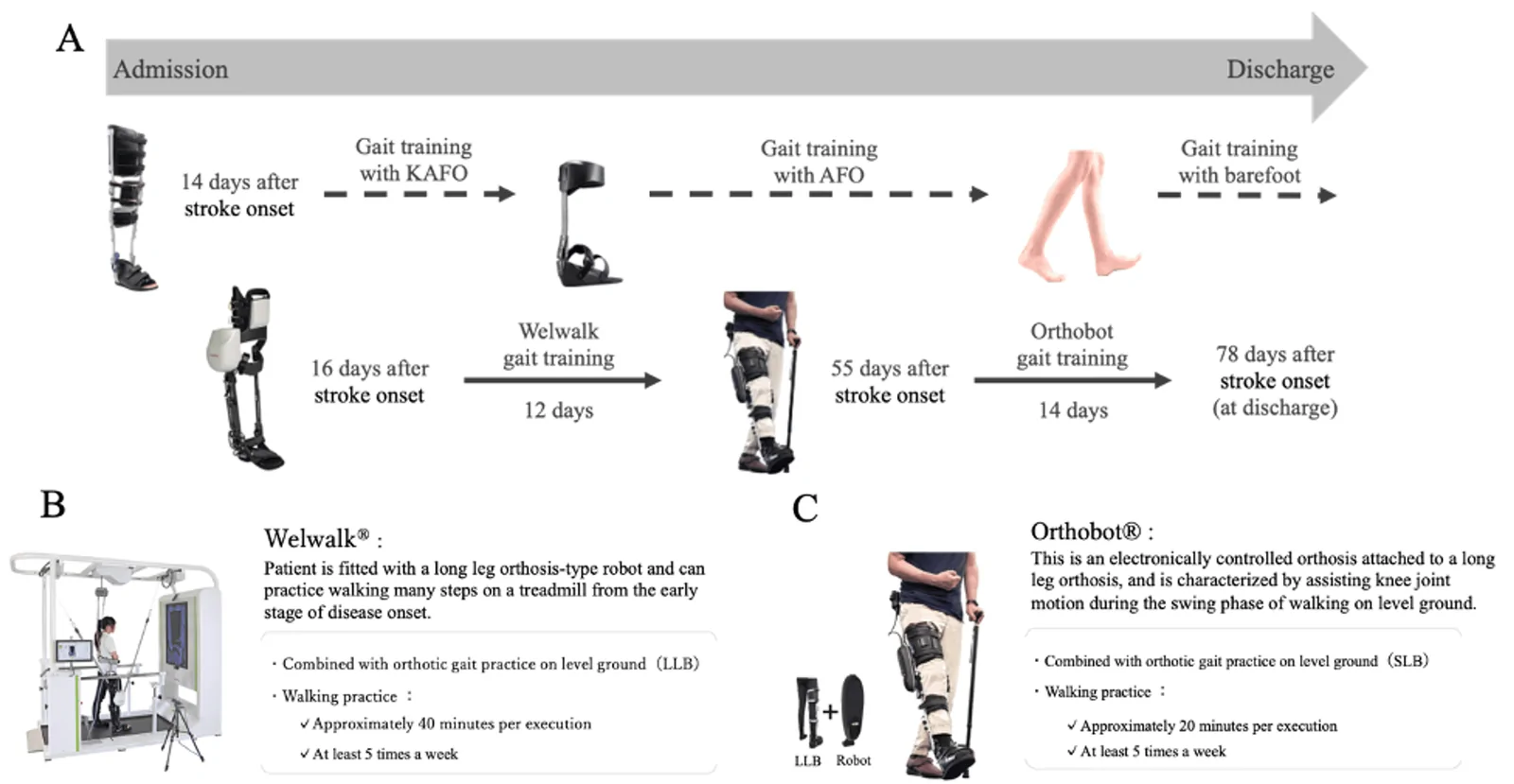
The intervention phases of the patient's gait training KAFO: knee-ankle-foot orthosis; AFO: ankle-foot orthosis Panel A: Schematic overview of the patient's gait-training course from admission to discharge. Conventional gait training progressed from walking with a KAFO, to walking with an AFO, and finally to practical barefoot gait training. Robot-assisted gait training was incorporated at specific stages according to the therapeutic goals: Welwalk®-assisted treadmill gait training was performed from days 16 to 28 after stroke onset during the early recovery phase, and Orthobot®-assisted overground gait training was performed from days 55 to 69. Panel B: Welwalk®, a treadmill-driven robot-assisted gait-training system used from days 16 to 28 after stroke onset. The patient walked on a treadmill while wearing a robotic long-leg orthosis, with adjustable assistance for paretic-limb swing and knee extension. In this case, Welwalk® was used primarily to provide intensive and repetitive gait practice aimed at improving walking independence during the early recovery phase. Panel C: Orthobot®, an electronically controlled robotic unit attached to a conventional KAFO. It was used from days 55 to 69 after stroke onset during overground walking to assist knee flexion and extension, particularly to facilitate knee flexion during the swing phase. In this case, Orthobot® was introduced after supervised walking had been achieved to address residual stiff-knee gait, reduced toe clearance, and compensatory movements. The Welwalk® image shown in panel B was reproduced with permission from Toyota Motor Corporation. The Orthobot® image shown in panel C was reproduced with permission from Suncall Corporation.

The walking intervention series is outlined as follows. During the early intervention phase, RAGT using a treadmill-driven robot (RAGT 1) was performed in parallel with long-leg brace walking practice on level ground, aiming to improve walking independence from day 16 after stroke onset. Specifically, continuous treadmill walking training was performed using a Welwalk® (Toyota Motor Corporation, Japan) (Figure 2B).

The Welwalk® enables a user to engage in treadmill-walking training from the early stages after stroke onset by wearing a long-leg orthosis-type robot. In the present case, the patient's walking practice with a Welwalk® was conducted for approximately 40 min/session, ≥5×/week, by physical therapists who had taken Welwalk® instructor training and were proficient in using Welwalk®. The device used in this case was the Welwalk® WW-1000 [10]. At the beginning of Welwalk® training, the swing assistance level was set to 2, the knee-extension assistance level to 7, the target knee-flexion angle to 40°, and the knee flexion-extension time to 0.86 s. No body-weight support was used, and the treadmill speed was 0.5 km/h. By the final phase of the Welwalk® intervention, the swing assistance level had been reduced to 1, while the knee-extension assistance level was adjusted between 1 and 2 according to the patient's gait performance. The target knee-flexion angle was increased to 60°, the knee flexion-extension time was adjusted to 0.94 s, and the treadmill speed was increased to 1.8 km/h. Body-weight support was not used throughout the Welwalk® intervention.

At the beginning of the intervention, the patient walked using the handrail and received physical assistance from a therapist for weight shifting. Around day 7 of Welwalk® training, therapist assistance was no longer required, and training was performed under supervision. Around day 10, the patient progressed from handrail-supported walking to walking with a treadmill cane. These settings were adjusted iteratively according to the patient's gait performance and level of therapist assistance rather than according to predefined numerical progression criteria.

As the patient's walking function improved, the degrees of freedom of the lower-limb joints of the orthosis were gradually adjusted, aiming for a transition to a short-leg orthosis.

After the general gait training phase (days 29-54 after stroke onset), the patient's level walking remained at the supervised level (FAC: 3) [11]. Immediately before the introduction of the orthosis-mounted robot (around day 55 after stroke onset), he continued to exhibit insufficient knee flexion of the paretic limb during the swing phase, consistent with a stiff-knee gait pattern, together with reduced toe clearance and associated compensatory movements. As these gait abnormalities had not sufficiently improved with general gait training, another phase of RAGT using an orthosis-mounted robot (RAGT 2) was introduced in parallel with orthosis walking practice. Specifically, the patient performed continuous walking practice on level ground with the use of an Orthobot® (Suncall Corporation, Japan) to facilitate knee flexion during the swing phase and to promote a lower-limb swing strategy with reduced reliance on compensatory movements (Figure 2C). The Orthobot® is an electronically controlled orthosis attached to a long-leg orthosis, and it focuses primarily on assisting its user's knee joint movement during the swing phase of level ground walking. The Orthobot® was used in combination with the short-leg orthosis walking practice on level ground, administered at a frequency of approximately 20 min/session, ≥5×/week. The Orthobot® parameters were adjusted according to the patient's walking speed and gait performance, with particular emphasis on assisting knee flexion during the swing phase. At the beginning of training, the knee-flexion assistance level was adjusted between 5 and 6 with the timing set between −2 and −3, while the knee-extension assistance level was 4 with the timing between −1 and −2. By the final phase, both assistance levels had been reduced to 2, with the knee-flexion timing adjusted between −1 and −2 and the knee-extension timing between 0 and −1.

During comfortable overground walking, assistance levels and timing were adjusted based on visual gait observation and the real-time lower-limb movement information displayed by the Orthobot®. Because knee flexion during pre-swing to initial swing was insufficient and delayed, flexion assistance was timed to facilitate earlier knee flexion, whereas extension assistance was adjusted to facilitate knee extension toward terminal swing and initial contact. These parameters were adjusted iteratively according to the observed gait pattern rather than predefined numerical progression criteria. Gait training was provided from days 14 to 77 after stroke onset, the day before the patient's discharge. The progression was as follows: From days 16 to 28 after stroke onset, treadmill-based RAGT was conducted as the Welwalk® gait training phase. From days 29 to 54 after stroke onset, general walking practice on level ground was performed (the general gait training phase). Finally, from days 55 to 69 after stroke onset, robotic gait training using orthoses was conducted (the Orthobot® gait training phase). After the completion of the Orthobot® intervention on day 69 after stroke onset, the focus of gait training shifted from robot-assisted gait-pattern correction to discharge preparation, including more practical walking practice reflecting situations expected in daily life after discharge. This training was continued until day 77 after stroke onset.

Evaluations

The Patient's Physical Function

The assessment of the patient's physical function included the lower-limb motor function items from the SIAS (i.e., the hip-flexion test, knee-extension test, and foot-pat test), the Modified Ashworth Scale (MAS) for the triceps surae, the 10-meter walk test (10MWT), the FIM motor and cognitive scores, and the FAC [11]. Isometric knee extension strength was measured using a Mobie handheld dynamometer (MT-100 series; SAKAI Medical Co., Ltd., Japan). Measurements were performed with the patient seated at the edge of the bed with the hip and knee joints flexed to approximately 90°. The dynamometer pad was positioned at the distal lower leg and stabilized using a fixation belt. Two maximal voluntary isometric knee-extension trials were performed, and the higher value was used for analysis. Force was recorded in Newtons (N). This series of physical therapy evaluations was conducted at admission (day 14 after stroke onset) and at discharge (day 78 after stroke onset).

The Patient's Abnormal Gait Pattern

Regarding the assessment of the patient's gait, we evaluated not only gait independence but also abnormal gait patterns. Specifically, we performed a three-dimensional motion analysis with a Kinema Tracer® 3D Gait Analysis System (Kissei Comtec, Japan) and four charge-coupled device (CCD) cameras. The walking task involved walking at a comfortable speed on a treadmill. Handrail use was permitted according to the patient's walking ability, and handrail support was used during both the pre- and post-intervention assessments under comparable testing conditions. The sampling frequency was set to 60 Hz. One gait cycle was defined as the interval from initial contact of the observed limb to the subsequent initial contact of the same limb. Eleven gait cycles were analyzed at the pre-intervention assessment and 12 gait cycles at the post-intervention assessment. For the identification of body landmarks, a 30-mm-diameter colored marker was attached to each of the following locations: the left and right acromion, the iliac crest, both hip joints (at the lower third of the line connecting the anterior superior iliac spine and the greater trochanter), both knee joints (on the line passing through the center of the patella in the knee extension position, at a point corresponding to half the anterior-posterior diameter of the distal femur excluding the patella), the lateral malleolus, and the fifth metatarsal head. A total of 12 markers were applied.

Based on the patient's body landmarks obtained from each color marker, abnormal gait patterns were identified, and toe clearance was calculated. As indicators of toe clearance, we calculated the following for the patient's paralyzed lower limb during the swing phase: (i) the vertical displacement of the fifth metatarsal head (i.e., toe clearance) and (ii) the change in the straight-line distance between the hip joint and the fifth metatarsal head (i.e., limb shortening). Toe clearance was calculated as the vertical displacement of the fifth metatarsal head from mid-stance to mid-swing (mid-swing minus mid-stance). Limb shortening was calculated as the reduction in the straight-line distance between the hip joint and the fifth metatarsal head from mid-stance to mid-swing (mid-stance minus mid-swing).

These indices were calculated for each gait cycle, and the mean value across the analyzed gait cycles was used as the representative value for each assessment. Cycle-to-cycle variability was expressed as the standard deviation across the analyzed gait cycles.

The difference between these two indicators can be explained as follows: the foot-to-ground distance is interpreted as an indicator reflecting the sum of compensatory movements such as hip elevation and lower-limb swing, whereas the limb shortening represents the functional shortening effect of the lower limb, which is inherently required during the swing phase of a gait and is composed of hip flexion, knee flexion, and ankle dorsiflexion; it is considered an indicator of toe clearance [10].

To quantify abnormal gait patterns, 12 abnormal gait indices were calculated from the three-dimensional kinematic data using predefined algorithms for each gait abnormality [12]. The indices were expressed as standardized scores based on reference data from healthy adults, with a mean of 50 and a standard deviation of 10. Accordingly, scores of 70 and 30 represent values 2 standard deviations above and below the healthy mean, respectively, while scores of 80 and 20 represent values 3 standard deviations above and below the healthy mean. An index value was calculated for each gait cycle, and the representative value for each gait abnormality was obtained by averaging the most severe one-third of the cycle-specific values. For the present patient, we also calculated the displacement distance of the hip joint upward due to pelvic tilt (pelvic elevation), the displacement distance of the foot upward due to abduction (swinging motion or abduction gait), and the displacement distance of the contralateral hip joint upward (opposite-side lifting) as components of compensatory movements, determining the displacement distance for each and their total sum [12].

We next performed three-dimensional motion analysis immediately before the initiation of RAGT 2 (around day 55 after stroke onset; FAC: 3) and at the end of RAGT 2 (day 69 after stroke onset; FAC: 4) to quantitatively analyze changes in the patient's gait patterns.

Results

The Patient's Physical Function

The data in Table 1 summarize the changes in the patient's physical function from admission to discharge. The changes in the motor items of the SIAS revealed that the patient's results on the hip flexion test improved from 3 points to 4 points; those on the knee extension test improved from 2 points to 4 points; and the foot-pat test score improved from 1 point to 2 points, demonstrating improvement in voluntary movement of the paretic lower limb. Regarding the sensory items of the SIAS, the patient's superficial sensation remained unchanged from admission, whereas his deep sensation improved from 2 points to 3 points.

**Table 1:** Changes over time in the physical function of the patient, a 53-year-old man with left hemiplegia due to a right lacunar infarction SIAS: Stroke Impairment Assessment Set; Sensory-LE: lower-limb sensory score of the Stroke Impairment Assessment Set; MAS: Modified Ashworth Scale; Mini-BESTest: Mini-Balance Evaluation Systems Test; 10MWT: 10-meter walk test; LLB: long-leg brace; SLB: short-leg brace; FIM: Functional Independence Measure. The Mini-BESTest was not assessed at admission, and the 10MWT could not be performed because the patient required walking assistance.

	Admission (day 14 after stroke onset)	Discharge (day 78 after stroke onset)
・SIAS		
Hip-flexion test	3	4
Knee-extension test	2	4
Foot-pat test	1	2
・Sensory-LE		
Surface	3	3
Deep	2	3
・MAS		
Triceps surae (0-4)	1	1+
・Knee extension strength (N)		
Paralyzed side	168.5	258.9
Unaffected side	412.6	442.6
・Balance function: Mini-BESTest (0-28)	-	26
・10MWT (sec)	-	8.99
・Walking style (parallel bars/Q-cane/T-cane/unaided walk)	Parallel bars	Unaided walk
・Orthosis (LLB/SLB/free)	LLB	Free
・FIM-mobility (score)	1	7
・FIM-motor (score)	33	85
・FIM-cognition (score)	23	35

On the MAS, the triceps surae value changed from 1 to 1+, indicating increased abnormal muscle tone. The knee extension muscle strength on the paralyzed side increased from 168.5 N to 258.9 N. On the unaffected side, knee extension muscle strength increased from 412.6 N to 442.6 N. At discharge, the patient's Mini-BESTest total score was 26 points. At admission, the patient wore a lower-limb orthosis and required walking assistance, making the measurement of his 10MWT time impossible. At discharge, his 10MWT time was 8.99 seconds during independent barefoot walking. Improvements were also observed in the patient's gait pattern, orthosis usage status, FIM motor score, and FIM mobility score.

The Patient's Gait Performance

Figure 3 illustrates the temporal changes in the patient's walking performance. At the initial evaluation (day 14 after stroke onset), the patient's moderate motor paralysis (SIAS-LE: 3-2-1) resulted in an assisted walking level (FAC: 1). During RAGT using the treadmill-driven robot, initiated on day 16 after stroke onset, the patient reached a supervised walking level (FAC: 3) by day 28 after stroke onset (RAGT 1 phase). The subsequent flat-ground walking practice showed stagnation in the patient's progression toward independent walking (general gait training phase). After the initiation of RAGT using the orthosis-mounted robot on day 55 after stroke onset, the patient's walking independence improved to FAC 4 by day 69 (RAGT 2 phase). Thereafter, during practical gait training, the patient progressed further and achieved FAC 5 by discharge on day 78.

**Figure 3:**
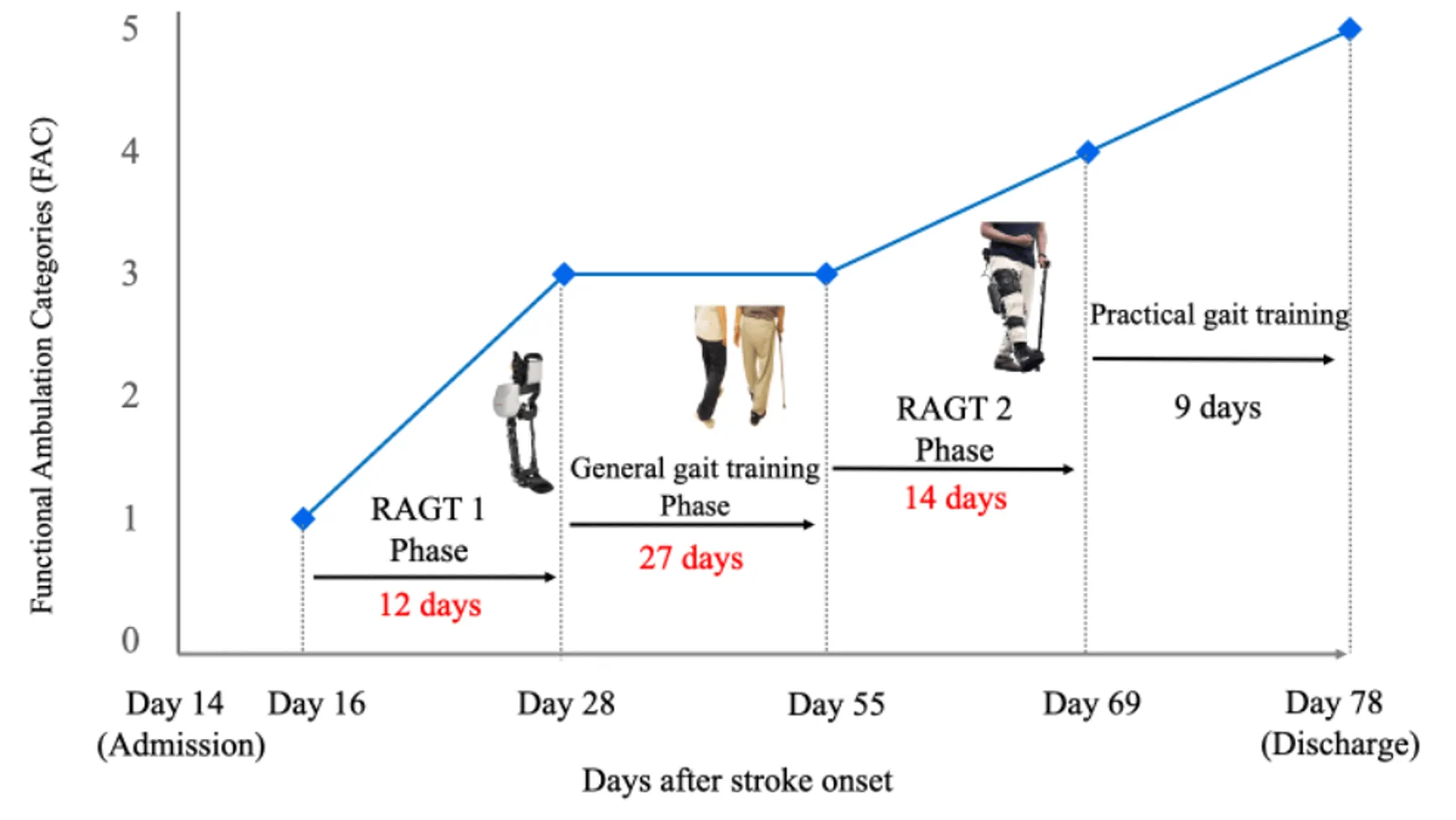
Changes in the patient's FAC scores and gait-training phases over time FAC: Functional Ambulation Category; RAGT: robot-assisted gait training The Orthobot® image was reproduced with permission from Suncall Corporation.

The Patient's Abnormal Gait Pattern

Figure 4 depicts the changes in gait parameters before and after the Orthobot® intervention. In the pre-RAGT 2 phase, insufficient knee flexion during the swing phase was primarily observed on the patient's paralyzed side, and the toe clearance was reduced on his paralyzed side compared to the non-paralyzed side.

**Figure 4:**
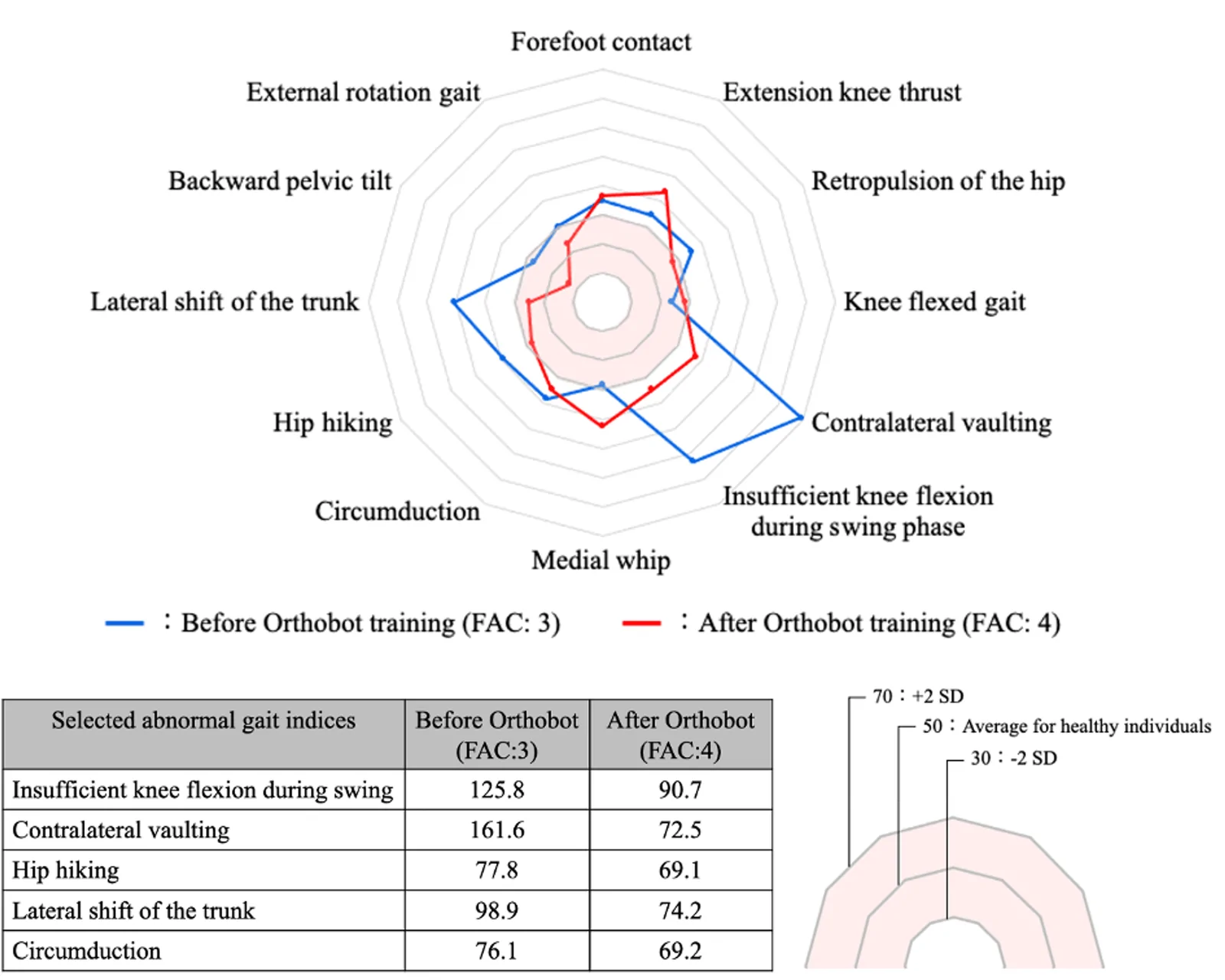
Changes in the patient's abnormal gait patterns before and after Orthobot® training FAC: Functional Ambulation Category; RAGT: robot-assisted gait training The radar chart shows standardized scores for 12 abnormal gait indices based on healthy reference data (mean=50; SD=10). A score of 50 represents the healthy reference mean, and the shaded area represents the healthy reference range of 30-70 (−2 to +2 SD). Scores outside this range indicate greater deviation from healthy gait. Numerical scores for selected abnormal gait indices are shown to facilitate direct comparison between assessments. In the post-RAGT 2 intervention phase, reductions were observed in the standardized scores for knee flexion deficiency during the swing phase and several compensatory gait indices.

Regarding changes in the toe clearance components, the limb shortening knee component increased, while compensatory components, primarily involving contralateral hip elevation, decreased (Figure 5).

**Figure 5:**
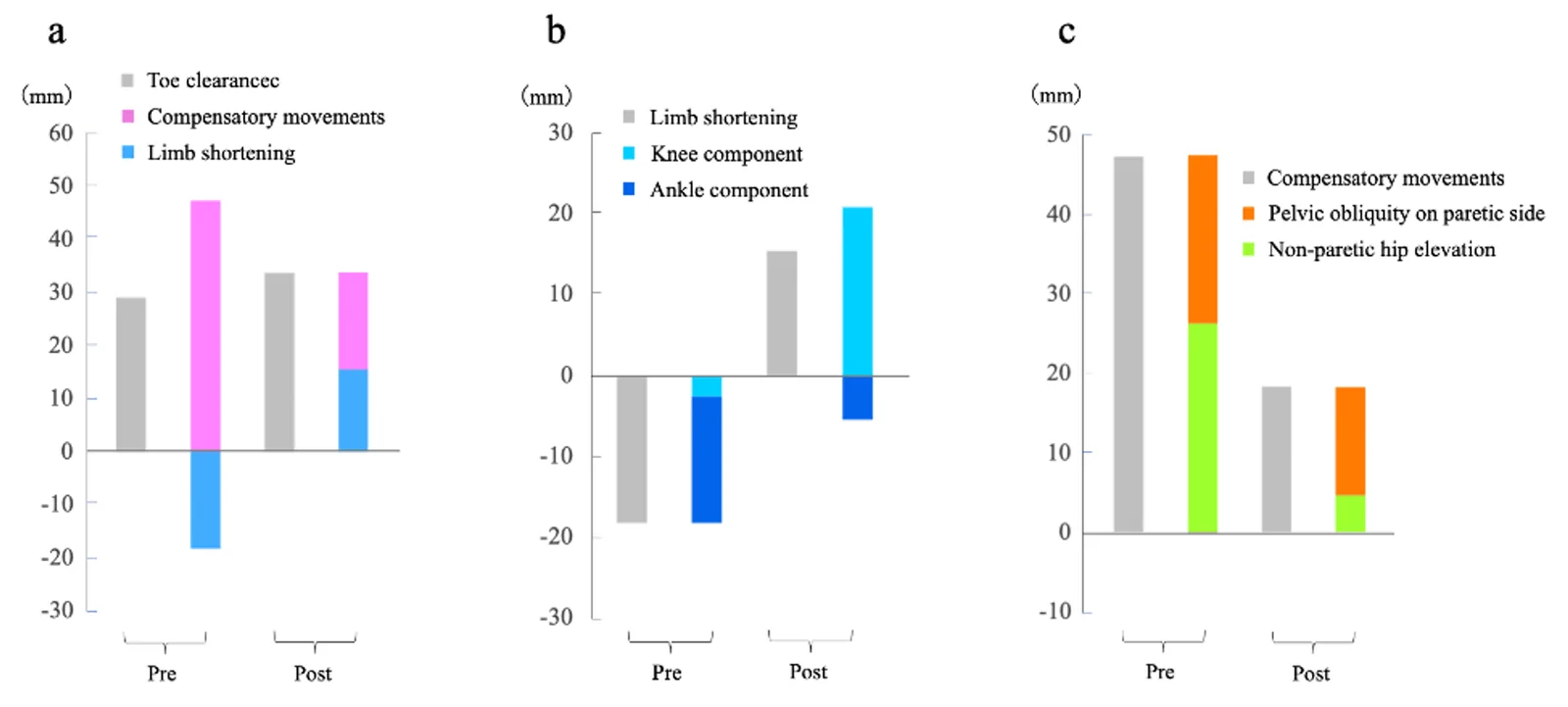
Changes in TC and its components before and after Orthobot® training TC: toe clearance; LS: limb shortening Panel A: Changes in TC and its components before and after Orthobot® training. TC is shown together with LS and compensatory movements to illustrate changes in the components contributing to foot clearance. Panel B: Changes in LS and its knee and ankle components before and after Orthobot® training. The panel illustrates changes in the joint-related components contributing to LS, particularly the increased knee-flexion component after the intervention. Panel C: Changes in compensatory movements and their components before and after Orthobot® training. The panel illustrates changes in compensatory strategies, including pelvic obliquity on the paretic side and non-paretic hip elevation. TC reflects the vertical displacement of the fifth metatarsal head during swing. LS represents the functional shortening of the lower limb produced by hip flexion, knee flexion, and ankle dorsiflexion, whereas compensatory components include pelvic elevation, circumduction, and contralateral hip elevation. Clearance-related indices were derived from the differences between mid-swing and mid-stance values; LS was expressed as the reduction in the distance between the hip joint and the fifth metatarsal head from mid-stance to mid-swing. The mean±SD of TC increased from 28.9±2.6 mm before the Orthobot® intervention to 33.8±3.3 mm after the intervention, while the mean±SD of LS changed from −18.7±2.1 mm to 15.3±4.3 mm. The knee-flexion component of LS increased, while compensatory components, primarily contralateral hip elevation, decreased after the intervention.

## Discussion

We examined changes in both walking independence and abnormal gait patterns during the sequential use of two types of RAGT according to a single patient's recovery process. During the early assisted-walking stage (FAC: 1), Welwalk®-based gait training combined with conventional orthotic gait training was associated with improvement in walking independence. Subsequently, after supervised level walking had been achieved (FAC: 3), Orthobot®-assisted gait training was associated with improvements in knee flexion during the paretic swing phase, increased toe clearance and limb shortening values, and reduced compensatory movements. Although spontaneous recovery and concurrent conventional rehabilitation may also have contributed to the observed improvements, the changes observed during each robotic intervention phase were consistent with the respective therapeutic goals. This intervention involved Welwalk®-based gait training starting on the third hospital day in the recovery ward (16 days after stroke onset), with stepwise adjustments to the level of assistance based on motor learning principles.

Regarding the effectiveness of treadmill-driven robots in improving walking independence, the authors of an investigation of hemiplegic patients with a first stroke who had an FIM gait score ≤3 points within 60 days of the onset of stroke reported that 40 minutes of walking practice per day using the Welwalk® device resulted in significantly higher walking independence compared to a group that only practiced walking with an orthosis [13]. It has also been reported that the degree of FIM improvement was significantly higher in the group of patients introduced to a treadmill-driven walking robot, indicating that improvements in walking independence were achieved with greater efficiency [14]. Similarly, in the present case, approximately 40 minutes of Welwalk® training performed at least five times per week was associated with improvement in walking independence. The clinical course was consistent with previous reports suggesting that treadmill-driven robotic gait training may contribute to improved walking independence [13,14].

On the other hand, immediately before the Orthobot® intervention (around day 55 after stroke onset; FAC: 3), the patient exhibited marked knee-flexion deficiency during the swing phase on his paretic side (stiff-knee gait), as shown in Figure 4. This stiff-knee gait is characterized by a decrease in peak knee flexion due to excessive knee extension moment during the swing phase, resulting in reduced toe clearance [15]. This is a representative abnormal gait pattern observed in approximately 25-75% of hemiplegic patients with gait impairment [16,17]. Consequently, this gait pattern promotes compensatory movements such as pelvic elevation and excessive pelvic rotation to compensate for the toe clearance deficit [7]. This not only reduces walking ability and increases energy expenditure but also heightens the risk of falling and acts as a barrier to achieving independent walking [17]. Indeed, in the present patient, stumbling due to insufficient knee flexion during walking was causing stagnation in his efforts to improve his walking independence.

To address this abnormal gait, we initially attempted correction through level-ground walking training combining visual and auditory feedback. In prior research, although visual and auditory feedback provided short-term improvements in spatiotemporal parameters, no group differences or consistent superiority were observed regarding the normalization of kinematic parameters or long-term transfer [18,19]. For example, it has been noted that even though visual feedback can result in visual symmetry in the step length, joint kinematic indicators and propulsive force asymmetry may persist [20]. Moreover, from a motor learning perspective, excessive reliance on external stimuli may impair error detection based on proprioception and the generalization of automation [21].

Indeed, in our patient's case, the changes did not persist when the feedback was removed, resulting in insufficient improvement in the stiff-knee gait. We therefore introduced an orthosis-mounted robot into conventional gait training to attempt to achieve the optimization of walking patterns (i.e., the Orthobot® gait training phase). Regarding changes in the toe clearance composition after intervention, although the patient's total limb shortening value increased, a reduction in compensatory components was observed, resulting in an overall increase in the toe clearance value. Fundamentally, toe clearance is an indicator that includes both normal limb shortening and compensatory movement elements, such as pelvic elevation, circumduction, and contralateral hip elevation. An increase in either limb shortening or compensatory components will thus relatively increase the toe clearance value. We interpret the improvement in the present patient's toe clearance values as a result of the acquisition of a gait pattern that is closer to normal, rather than to an increase in compensatory movement (Figure 5a). Regarding the changes in the composition of limb shortening in the present patient, we observed primarily an increase in the knee component. In normal gait, limb shortening consists of a compound movement involving hip flexion, knee flexion, and ankle dorsiflexion, with knee flexion contributing most significantly [15]. Conversely, the core pathology of the stiff-knee gait is excessive knee extension torque during the pre-swing and early swing phases. This torque limits the knee flexion angle and angular velocity, reducing the peak knee flexion value during the swing phase.

Previous research on an orthosis-mounted robot has suggested that mechanically assisting knee motion during the swing phase may facilitate knee flexion during gait [9]. After the Orthobot® phase, both swing-phase knee flexion and the knee-flexion component of limb shortening increased. These changes were consistent with the therapeutic goal of facilitating knee flexion during the swing phase. Furthermore, these changes may have contributed to the acquisition of a lower-limb swing strategy with reduced reliance on compensatory movements (Figure 5b). After the Orthobot® phase, treadmill-based gait analysis showed improvements in stiff-knee gait and toe clearance. These findings suggest that selecting robotic devices according to the patient's recovery stage and therapeutic goals may be a useful strategy for promoting both walking independence and gait-pattern optimization.

This case report and the intervention have several limitations to consider. First, as this intervention is a preliminary verification using a single case, the case characteristics and pathologies for which the RAGT strategy presented herein is applicable remain unclear. Second, the gait analysis in this report focused on one aspect of kinematic variables and did not consider the kinetic or electromyographic aspects. Although handrail support was used under comparable conditions in both gait assessments, its potential influence on swing-phase kinematics could not be separately quantified. Because gait analysis was conducted during treadmill walking, the observed kinematic changes cannot be assumed to represent changes during overground walking; transfer to overground gait was not directly assessed. Third, although assist levels and timing were adjusted based on clinical gait observation and device-provided feedback, these adjustments were not based on predefined quantitative criteria, and the optimal parameter-setting strategy remains unclear.

To address these limitations, future studies should expand the sample size and attempt more multifaceted quantitative gait analyses. These advances are necessary to design RAGT intervention strategies that are tailored to the specific pathophysiology of gait disorders.

## Conclusions

This case report describes the stepwise use of two types of RAGT according to the recovery stage of gait impairment after stroke. Treadmill-based RAGT was used in the early phase to promote walking independence, whereas orthosis-mounted RAGT was introduced after supervised level walking had been achieved to address residual abnormal gait patterns. The sequential approach was feasible in this case. The patient ultimately achieved independent walking and showed improvement in stiff-knee gait and toe clearance. These findings suggest that stage-specific selection of robotic devices may be useful for promoting both walking independence and gait-pattern optimization after stroke; however, the observed improvements were associated with, rather than solely attributable to, the sequential robotic intervention.
